# Co-expression network analysis of protein phosphatase 2A (PP2A) genes with stress-responsive genes in *Arabidopsis thaliana* reveals 13 key regulators

**DOI:** 10.1038/s41598-020-77746-z

**Published:** 2020-12-08

**Authors:** Zaiba Hasan Khan, Swati Agarwal, Atul Rai, Mounil Binal Memaya, Sandhya Mehrotra, Rajesh Mehrotra

**Affiliations:** 1 Department of Biological Sciences, K.K. Birla Goa Campus, BITS-Pilani, Goa, India; 2 Department of Computer Science and Information Systems, K.K. Birla Goa Campus, BITS-Pilani, Goa, India

**Keywords:** Computational biology and bioinformatics, Plant sciences, Plant stress responses

## Abstract

Abiotic and biotic stresses adversely affect plant growth and development and eventually result in less yield and threaten food security worldwide. In plants, several studies have been carried out to understand molecular responses to abiotic and biotic stresses. However, the complete circuitry of stress-responsive genes that plants utilise in response to those environmental stresses are still unknown. The protein phosphatase 2A (PP2A) gene has been known to have a crucial role in abiotic and biotic stresses; but how it regulates the stress response in plants is still not known completely. In this study, we constructed gene co-expression networks of PP2A genes with stress-responsive gene datasets from cold, drought, heat, osmotic, genotoxic, salt, and wounding stresses to unveil their relationships with the PP2A under different conditions of stress. The graph analysis identified 13 hub genes and several influential genes based on closeness centrality score (CCS). Our findings also revealed the count of unique genes present in different settings of stresses and subunits. We also formed clusters of influential genes based on the stress, CCS, and co-expression value. Analysis of *cis*-regulatory elements (CREs), recurring in promoters of these genes was also performed. Our study has led to the identification of 16 conserved CREs.

## Introduction

Gene co-expression network analysis (GCNA) has attracted a lot of interest recently in plants, and various methods have been introduced for their inference biologically and statistically from a large amount of gene expression data. Continuous exposure of plants to environmental stresses caused by drought, salt, cold, heat, osmotic stress, and biotic stresses hinders their growth and development and subsequently results in less yield, productivity, and threaten food security worldwide. Exposure to such stress conditions triggers an internal signaling cascade in plants to initiate a unique response to a particular stress or shared responses common to individual stresses. It has been reported previously that there is a significant overlap of expression of genes that provide defense against various abiotic and biotic stresses. A broad spectrum of genes commonly participating in responses to these stresses as shared or general stress-responsive genes has been identified. Several reports revealed cross-talk between these stress responses synergistically or antagonistically^1^.

Protein phosphatases are an important group of enzymes, which in combination with their counterpart protein kinases mediate diverse cellular processes in cells by dephosphorylation and phosphorylation respectively to their target proteins. In plants, two major types of Ser/Thr phosphatases are present based on their substrate specificity and pharmacological properties: Type 1 (PP1) and Type 2 (PP2). PP2 enzymes are further categorised into PP2A, 2B, and 2C, based on their requirement for divalent cations. PP2B is not identified in plants yet. While both the PP2A family and PP2C family are distinct from each other and present in plants as well as in animals. PP2A are trimeric type 2A protein phosphatases, composed of a scaffold subunit *A*, regulatory subunit *B*, and a catalytic subunit *C*. In the past few years PP2C and various subunits of PP2A from *Arabidopsis* and other plants have been cloned and characterised successfully in plants. These studies reveal the involvement of PP2A in various stresses such as heat^2^, salt^3^, oxidative stress^4^ while PP2C are involved in drought^5^, salt^6^. Earlier we have shown that the PP2C promoter and its variants from *Arabidopsis thaliana* are stress-responsive. They were found to be induced in response to ABA^7^. Regulatory subunit *B* are evolutionarily conserved signaling components that regulate stress signaling in both animals and plants^8^. Many studies have been carried out on kinase proteins so far but few on protein phosphatases in animals and plants. The specificity of PP2A towards its target is accomplished by the *B* subunit. Since PP2A is a diverse enzyme in nature it is known as an important regulator in a wide range of plant processes such as ROS signaling, light acclimation, biotic and abiotic stress like heat, salinity, drought^9^.

*Arabidopsis thaliana* has long been a popular model plant for basic biological research. The availability of huge amounts of transcriptomic data deposited in various databases, including data under certain environmental stress conditions made it feasible for us to construct co-expression networks to reveal the relationships between gene expression and stress regulation. Understanding gene regulatory networks primarily requires the discovery of expression modules within co-expression networks among genes, followed by the identification of motifs within promoters and their corresponding transcription factors that regulate expression. The co-expression network is based on correlations of expression values between two genes. Generally, it specifies correlation patterns between genes in a pairwise manner across multifarious samples of microarray/RNA-sequencing. Building and analysis of co-expression networks are crucial for inferring gene functions, their annotations, biological pathways in which the group of genes is involved, candidate disease gene prioritisation and the identification of regulatory genes during stress conditions by examining a huge collection of complex data^10^.

A gene co-expression network (GCEN) is a collection of genes in a cell that interacts with each other. A GCEN is normally represented as an undirected graph, where a vertex (node) represents a gene and an undirected edge represents a significant co-expression relationship between the genes considering a series of gene co-expression measurements. A module extracted from a co-expression network may contain co-regulated gene clusters that interact among themselves and take part in a common biological process^11^. Microarray analysis is one of the crucial techniques to measure the expression of different genes simultaneously in a plant cell and provide functional data for those genes. However, not many studies have investigated co-expression networks of PP2A in plants during stress conditions using microarray data. Therefore, it is a prerequisite to select corresponding genes related to stress conditions from thousands of genes with the help of appropriate in-silico approaches or other computing measures such as distribution pattern or graph analysis.

One of the crucial levels of regulation of gene expression is transcription. It is one of the tightly controlled processes in a plant cell which is reflected in the percentage of the *Arabidopsis* genome dedicated to transcription factors only (approximately 5% of genome coded for 1533 TFs)^12^. Cooperate binding of transcription factors (TFs) with specific *cis*-acting DNA elements is essential to regulate the time and space-specific expression of genes in each cell and organs in plants during stress^13^. Comprehensive knowledge about how such CREs function and novel discoveries of CREs are crucial for identifying the mechanism by which cells sense and correctly reciprocate to their environment when exposed to any stress.

We performed Co-expression network analysis followed by CRE or motif analysis^14^.Our method first builds a co-expression network of PP2A genes from *A. thaliana* and further searches for *cis*-regulatory elements enriched within the promoters of each gene present on the network. The notion behind that if two genes share common *cis*-regulatory elements, they are likely to be involved in the same function, which also shows the functional relevance of genes.

Gene co-expression network analysis has been popular in *Arabidopsis thaliana* during single stress like salinity stress^15^ or combined stresses such as salt, heat, cold, light^16^. Studies conducted on maize^17^ and *Arabidopsis*^18^ identified various gene modules in stress responses pathways. Huang *et al*^19^ identified modules followed by hub gene identification in *Arabidopsis* under light response. A meta-analysis in *Arabidopsis* plant unveils how perturbations preferentially affect connected, central and organised genes in modules of network^20^. Gene networks are rapidly used in economically important plant species aimed to study modules and identification of hubness since the past two years for growth and development in Bamboo^21^, stress responses in cotton^22^, graft healing in tomato^23^, flower and fruit development in wild strawberry^24^, plant height development^25^ and salt and drought stress^26^ in maize, identification of drought-responsive genes in rice^27^, Theanine biosynthesis in tea^28^, sesame gene functions^14^.

## Results

### Identification of Hub genes

**Figure 1:**
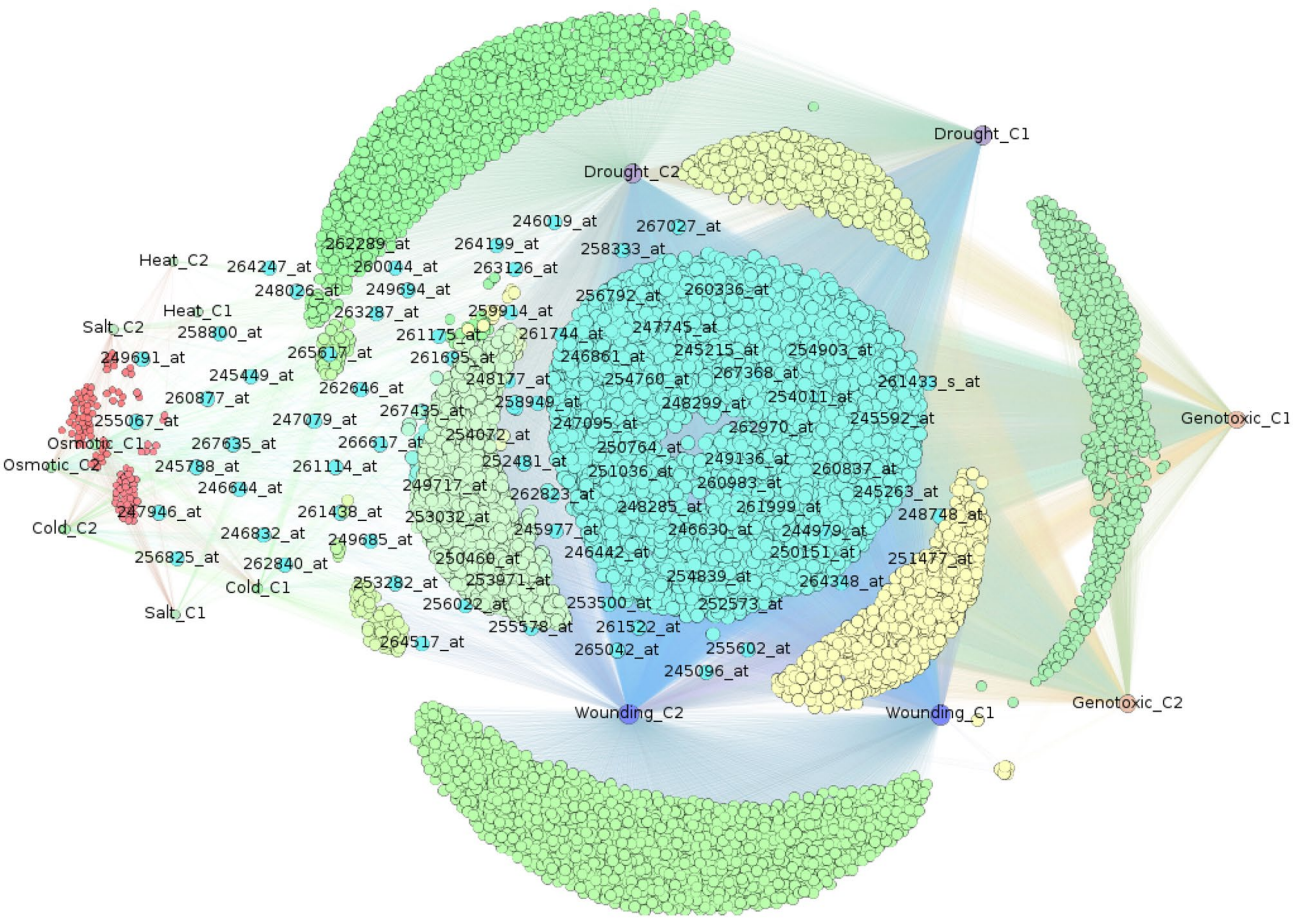
Graph analysis of genes present in C subunit across all stresses. Nodes represent two sets- stress condition and subunit pair ($C_1$, $C_2$). Edges represent the occurrence of a gene in stress-subunit pair. Edge weight represents the co-expression score. Size of the node represents the relative closeness centrality score. Colour represents the group of nodes with same closeness centrality score.

Hub genes are those genes that have a high degree of connectivity in terms of their co-expression values. The genes for PP2A subunits (A, B, C), obtained from the database under stress condition, were used in order to identify the hub genes through graph analysis on the gene network. We select all those genes whose co-expression value is equal or above mean score (refer to section "Materials and methods"). We use the closeness centrality metric to represent the closely connected or influential genes in the network. Supplementary Figs. S2, S3, and manuscript Fig. 1 illustrate the gene network constructed for A, B, and C subunits, respectively. The nodes represent the gene ids, stress and unit pairs ($SU_{i,j}$) and the edges represent the occurrence of gene ids in respective stress and unit pair. In supplementary Figs. S2, S3, and manuscript Fig. 1, the first set of $SU_{i,j}$ had a total of 35, 70, and 14 nodes in *A*, *B*, and *C* subunits, respectively (7 stresses condition multiplied by the number of genes forming that subunit). Whereas, the second set of gene ids in *A*, *B*, and *C* network had 20,305, 16,497, and 10,276 unique nodes.

**Figure 2:**
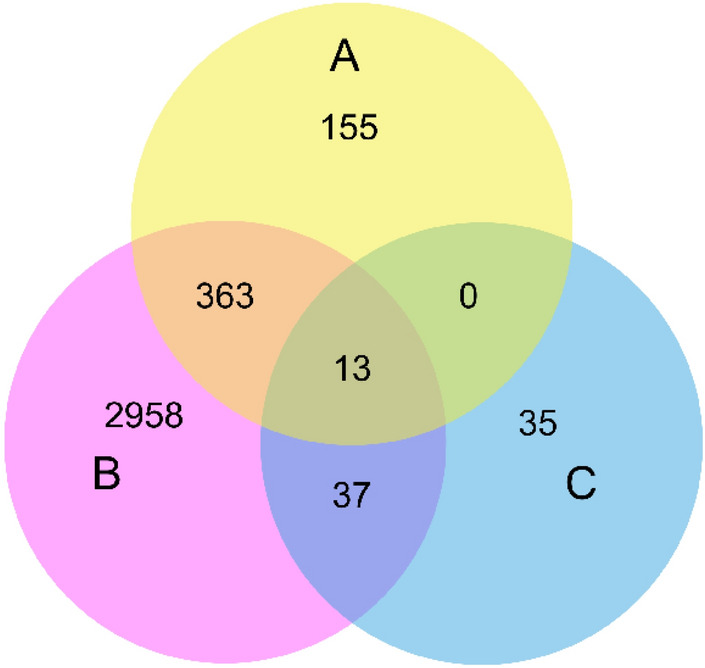
3-set Venn diagram illustrating the count of unique and common genes in each possible pair of A, B, and C Subunits. The 13 genes identified from $A \cap B \cap C$ are the hub genes used for motif analysis.

Due to a large number of nodes in *A* subunit, the network graph (Supplementary Fig. S2) has resulted in a dense graph. Subunit *B* had a slightly lesser number of unique genes but a large number of nodes had similar coexpression scores (above threshold) resulting in large number of edges in the network. Supplementary Fig. S3 shows the circular and non-overlapping layout of genes arranged counter-clockwise in the increasing order of their closeness centrality score (CCS). The genes having similar scores end up at the same angle in the circle. The number of genes in subunit *C* is almost $50\%$ of subunit *A* with a co-expression value above the mean score (refer to section "Materials and methods"). Figure 1 shows a clear distinction of the genes along with stress-unit pairs. For better readability of the ids, we scale the node size and colour proportional to the centrality score. The higher the node size, the higher the closeness centrality score. Furthermore, we chose “Fruchterman-Reingold graph drawing” layout which dissuades the hubs and prevents overlapping of gene ids^29^. Fruchterman-Reingold graph drawing algorithm was proposed by Thomas M. J. Fruchterman, first published in 1991 for drawing undirected graphs with straight edges^30^. The nodes having similar behaviour in-terms of CCS are pushed to the same side of the graph. We further apply “Label Adjust” layout to avoid the overlapping node labels^31^. Label Adjust algorithm is primarily used in conjugation with other layout algorithms to first find the layout and then adjust the labels in the resulting layout. The graph analysis reveals the set of $SU_{i,j}$ that has maximum connectivity in the network and hence reduces the size of the network diameter and make it more connected. However, not all stresses have a large number of genes with above mean co-expression value. Hence, their node size is smaller than other highly connected stress conditions. For example, in *A* subunit, unlike heat stress condition, wounding, genotoxic, and cold stress conditions have high closeness centrality. Similarly, in *C* subunit, wounding, genotoxic, and drought have highest centrality scores but osmotic, cold, salt and heat have very low centrality scores. This reveals that the stress conditions with low centrality scores have very less number of genes with high (above mean) co-expression value. As represented with colours and node size, many genes in each of these subunits have similar centrality scores. Therefore, we enforce a threshold (significant decline in the centrality) and display the names of only those genes that have centrality equal or above the threshold. We, however, display the names of all stress conditions to visualise their overall connectivity in the network.

Figure 2 shows a Venn diagram of common genes in each subunit with high CCS (the independent threshold for each subunit). Figure 2 reveals that *A*, *B*, and *C* subunits have 531, 3371, and 85 genes with high CCS, respectively. The pair of (*A*, *B*) and (*B*, *C*) have 363 and 37 common genes, respectively. *C* and *A* do not have any gene ids that are common between them but not with *B* subunit. Venn diagram shows that among all genes used for graph analysis, only 13 genes with high centrality score are common across all subunits. We performed GO term analysis on these genes using the DAVID tool^32^, which shows that, out of 13 genes, 9 genes are involved with chloroplast function. The main function of chloroplasts is performing photosynthesis, but they also affect the physiology, growth and development since they are involved in the synthesis of amino acids, nucleotides, fatty acids, phytohormones, and vitamins. Metabolites synthesised in chloroplasts protect plants from abiotic stress such as cold, drought, heat, salt, light, and biotic stresses, pathogens. Several studies show that being photosynthetic organelles, chloroplasts are highly sensitive towards heat stress, which directly or indirectly affects photosynthesis, chlorophyll biosynthesis, photochemical reactions, electron transport, and CO2 assimilation. Table 1 shows the CCS, gene name and gene function of these 13 genes. Table 1 unveils that all the 13 PP2A genes identified as common across all subunits have exactly the same centrality in their respective subunits, i.e. connected with (or present in) an equal number of stress conditions.

**Table 1:** Common PP2A gene ids across all subunits with high centrality scores along with their gene name and function.

Gene id	A	B	C	Gene name	Gene function
258949_at	0.50	0.47	0.50	DCL PROTEIN	Chloroplast organisation, chloroplast rRNA processing
258800_at	0.50	0.47	0.50	TRANSMEMBRANE PROTEIN	–
258333_at	0.50	0.47	0.50	PLASTOGLOBULAR PROTEIN 18, PG18	Plastoglobular protein which is involved in chloroplast function and thylakoid formation
256825_at	0.50	0.47	0.50	ZINC FINGER PROTEIN-LIKE PROTEIN	Regulation of transcription
256792_at	0.50	0.47	0.50	MAR BINDING FILAMENT-LIKE PROTEIN 1, MFP1	Encodes a DNA-binding protein that binds to plastid DNA non-specifically and is associated with nucleoids and thylakoid membranes
254760_at	0.50	0.47	0.50	CHLOROPLAST IMPORT APPARATUS 2, CIA2	Transcription regulator responsible for specific upre/gulation of the translocon genes atToc33 and atToc75 in leaves. Involved in protein import into chloroplast
253971_at	0.50	0.47	0.50	ARABIDOPSIS THALIANA CRM FAMILY MEMBER 2, ATCFM2, CFM2, CRM FAMILY MEMBER 2	Encodes a protein containing CRM domain that is involved in group I and group II intron splicing
252481_at	0.50	0.47	0.50	CELL WALL-PLASMA MEMBRANE LINKER PROTEIN, CWLP	–
249685_at	0.50	0.47	0.50	IAA-ALANINE RESISTANT 2, IAA28, IAR2, INDOLE-3-ACETIC ACID INDUCIBLE 28	Negative regulator of lateral root formation in response to auxin
248177_at	0.50	0.47	0.50	RAF1, RUBISCO ACCUMULATION FACTOR 1	Rubisco biogenesis
247946_at	0.50	0.47	0.50	AEF1, ATPF EDITING FACTOR 1	Involved in RNA editing of plastid atpF and mitochondrial nad5
246861_at	0.50	0.47	0.50	DCL protein (DUF3223)	Chloroplast rRNA processing
246630_at	0.50	0.47	0.50	CCB3	Cofactor assembly, complex C (B6F)(CCB3)

**Figure 3:**
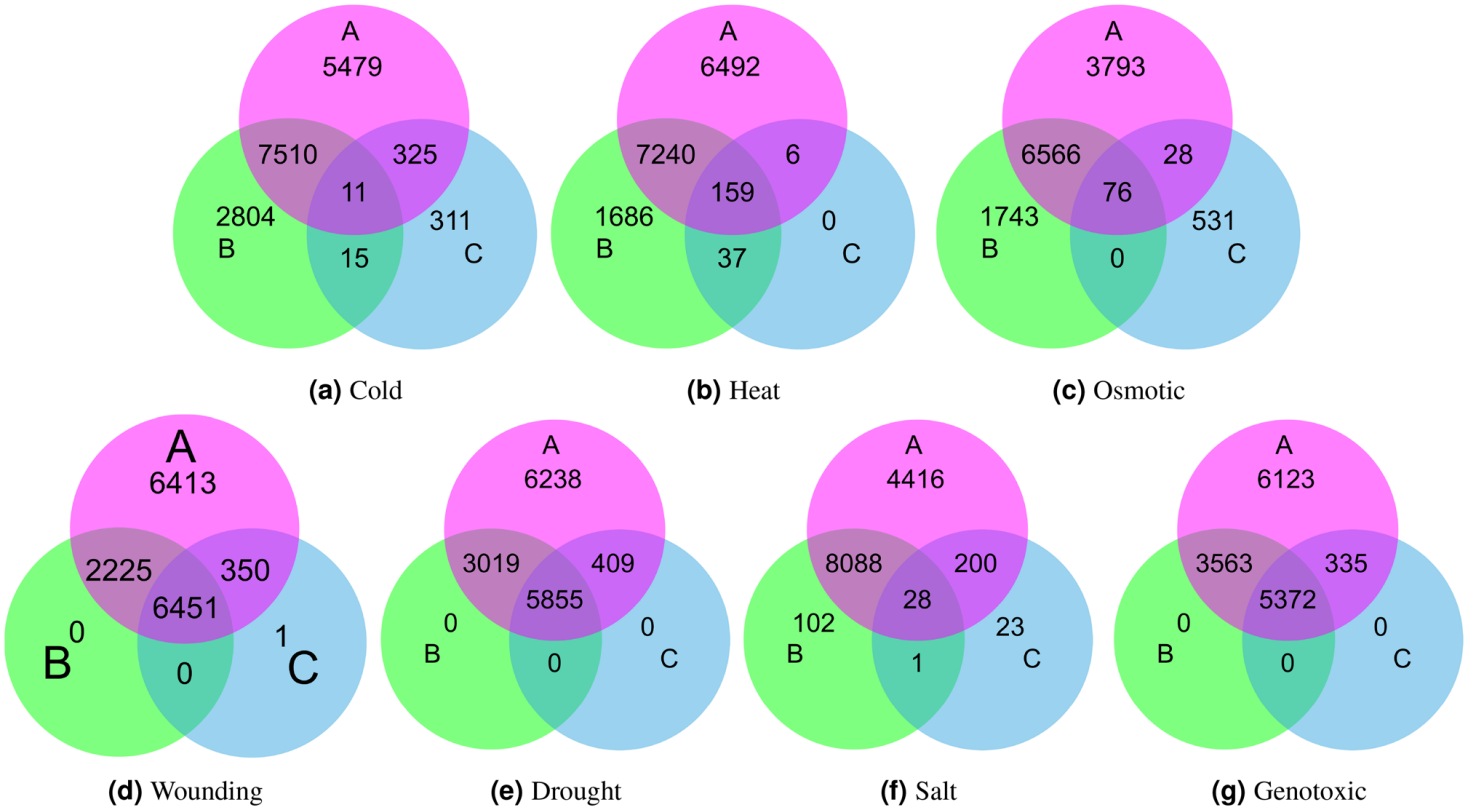
Venn diagram of gene ids extracted after graph analysis. The venn diagrams represent the count of common and unique genes in A, B, and C subunits in different stress conditions.

**Figure 4:**
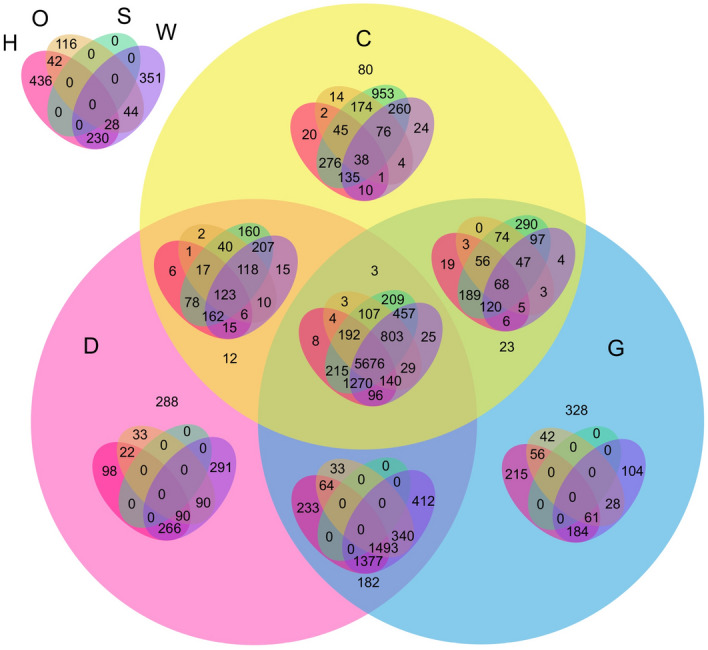
7-set Venn diagram of gene ids present in A subunit across all stress conditions. H: Heat, O: Osmotic, S: Salt, W: Wounding, D: Drought, C: Cold, G: Genotoxic. The 7-set venn diagram illustrates the count of genes in all 127 possible combinations.

**Figure 5:**
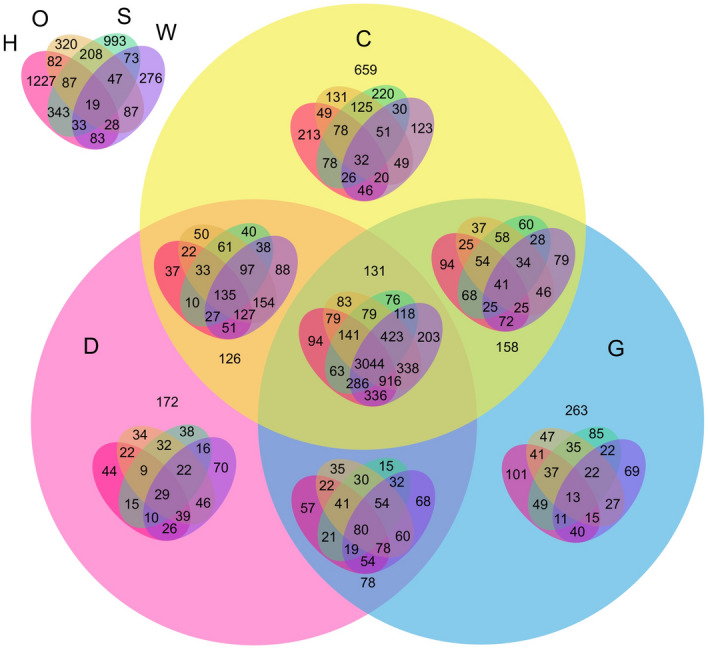
7-set Venn diagram of gene ids present in B subunit across all stress conditions. H: Heat, O: Osmotic, S: Salt, W: Wounding, D: Drought, C: Cold, G: Genotoxic. The 7-set Venn diagram illustrates the count of genes in all 127 possible combinations.

**Figure 6:**
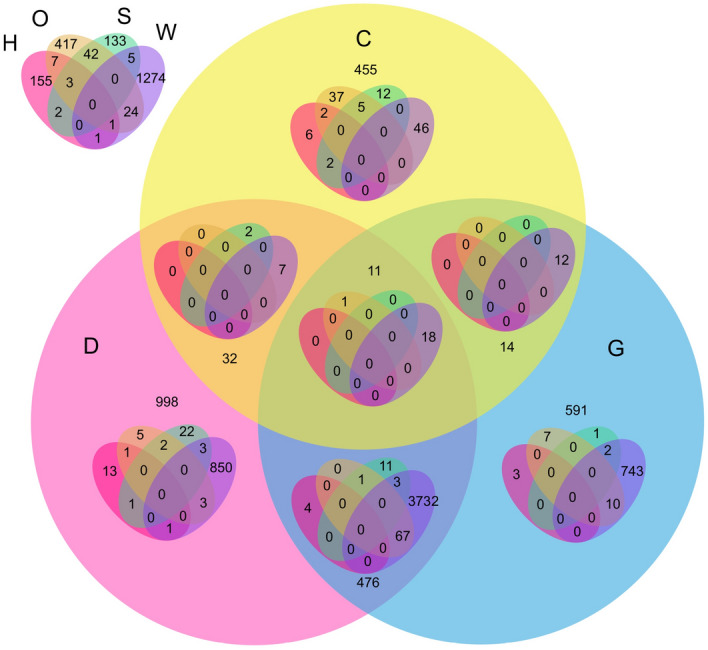
7-set Venn diagram of gene ids present in C subunit across all stress conditions. H: Heat, O: Osmotic, S: Salt, W: Wounding, D: Drought, C: Cold, G: Genotoxic. The 7-set Venn diagram illustrates the count of genes in all 127 possible combinations.

In addition to the high CCS genes intersection, we also perform an analysis of all ids used in graph analysis in independent stress conditions. Figures 3 depicts the distribution in independent subunits and number of common genes in 3-set Venn diagrams. As shown in Figure 3a, within cold stress there are 11 genes common across all subunits (AT4G34120, AT2G40880, AT1G14120, AT4G01450, AT4G35020, AT5G25900, AT1G14340 , AT1G14340 , AT2G39700, AT3G48750, AT4G12400). GO term analysis reveals that most of the genes were found to be involved in the biological process and in the cell component. Three genes: AT4G35020, AT3G48750, AT4G12400 were overrepresented with the term cytoplasm whereas 2 genes: AT5G25900, AT4G35020 were involved in KEGG pathways. Figure 3b unveils that in heat stress condition, all the genes present in *C* subunit are also present in either *A* or *B* or both *A* and *B*. There are six genes (AT1G80720, AT5G07290, AT2G34260, AT4G32960, AT1G78240, and AT3G56120) which are present in $A\cap C$; 37 in $B\cap C$; and 159 are present in $A \cap B \cap C$. All the six genes are part of the cell component and involved with the function in nucleus, mitochondria and golgi. Figure 3c shows that in osmotic stress condition, all the genes that are present in both *B* and *C* subunits are also present in *A* subunit; While *A* has genes common with *B* (6566) and *C* (28) individually. These graphs also show the overall distribution of genes across each subunit in various stress conditions. For example, *C* has the least number of genes with above-average co-expression value. Figures 3e,g reveal that in drought and genotoxic stress conditions, *B* and *C* have no unique genes, and all of them are common with *A* subunit; while in wounding stress condition (Fig. 3d, only one gene is unique in *C* subunit i.e AT5G10300. This gene is biotic stress responsive, found to be involved with jasmonic acid (JA) and salicylic acid (SA) responses. Due to which the common genes across all subunits are very high in comparison to others. As discussed in Section Co-expression network construction and parameters, salt and drought have a similar distribution of co-expression values. However, in Fig. 3e,f, Venn diagrams show that despite having similar co-expression scores, the genes do not have similar patterns in occurrences in different subunits. For example, in drought conditions, *B* and *C* do not have any unique ids, and all of them are present in *A* subunit. Whereas, in salt condition, *B* and *C* have unique genes as well as the total number of genes present across all subunits are significantly smaller than drought conditions. Further, *B* and *C* have one gene common which is not present in *A* subunit, i.e. AT1G27050. This gene is part of the nucleus associated with the transcription process in the cell.

In contrast to the prior literature, we also conduct an analysis on the genes present in different stresses in independent subunits. We conduct this analysis to support our study of Co-expression behaviour of genes not only within different subunits but also within different stresses of the same subunit. We used VennPainter^33^, an open-source tool for drawing Venn diagrams with nested venns upto seven sets. Figures 4, 5, and 6 illustrate the 7-set Venn diagrams created for all stresses in *A*, *B*, and *C* subunits respectively. Figures 4, 5, and 6 unveil that analysing seven sets for finding common genes is challenging ($2^n -1$ = $2^7 - 1$ = 127) possible combinations, where *n* is the number of sets. For better readability of Venn diagrams, we use short labels for stresses, i.e. Heat (H), Osmotic (O), Salt (S), Wounding (W), Cold (C), Drought (D), and Genotoxic as (G). The interpretation of these Venn diagrams is done as follows: the Venn diagram is divided into two parts; 3-set (C, D, G) and 4-set (H, O, S, W). The first set represents the number of unique and common genes in C, D, G, C $\cap$ D, C $\cap$ G, D $\cap$  G, and C $\cap$  D $\cap$ G. Whereas, the second set represents the unique genes in 24 combinations in H, S, O, W, H $\cap$  S, H$\cap$O, $\cap$, S$\cap$ O, S $\cap$ W, O $\cap$ W, H $\cap$ S $\cap$ O, H $\cap$ S$\cap$ W, H $\cap$  O $\cap$ W, S $\cap$O $\cap$  W, H $\cap$  S $\cap$ O $\cap$ W. Placing the 4-set Venn diagram in each intersection of the circle gives remaining statistics. For example, placing HSOW: H $\cap$ S $\cap$ O $\cap$ W set in single unit gives 5-set results- HSOW with D, C, G. Similarly, 2-set intersection reveals insights from 6 sets; HSOW with C  $\cap$ D, C $\cap$ G, D $\cap$ G. Placing HSOW Venn diagram in the final intersection cell of C $\cap$ D $\cap$ G gives the statistics on 7-set.

Figure 4 reveals that within *A* subunit, 5676 genes are present across all stresses. There are no unique genes present in Salt stress which are not present in any other stresses. Figure 4 reveals that drought, cold, and genotoxic have three gene ids in common (AT1G12780, AT3G27230, 254337_at (TAIR id is not available)) which are not present in any other stress. AT1G12780, AT3G27230 genes are part of the cell component concerned with golgi apparatus. The set of cold, genotoxic, drought, and osmotic have three genes (AT4G17940, AT4G35690, AT2G37970) in common which are not present in the remaining three stress conditions. AT4G17940, AT4G35690 were found to be over-represented with the term chloroplast which means that they are involved in functions related to chloroplast. Figure 5 reveals that unlike subunit *A*, subunit *B* has 993 gene ids which are unique in salt stress but not present in any other stress. Despite having a larger set of genes in *B* subunit, the number of common genes across all stresses are significantly smaller (3044) than *A* subunit. We found an interesting insight from Fig. 5, that in the *B* subunit, each combination out of 127 has some unique genes which are not present in any other combination (intersection). The smallest number of common genes (AT1G11470, AT4G27620, AT3G24440, AT1G70590, AT1G62410, AT2G01820, AT1G58070, AT4G12840, AT5G62640) are present in the $\{Drought \cap Heat \cap Osmotic \cap Salt\}$ followed by 10 genes (AT5G51550, AT1G61640, AT4G31790, AT1G21840, AT3G55510, AT5G56580, AT3G14020, AT1G70530, AT2G31400, AT5G35430) in $\{Heat \cap Salt \cap Drought \cap Cold\}$. AT1G11470, AT4G27620, AT3G24440, AT1G62410, AT2G01820, AT1G58070, AT4G12840, AT5G62640 were found to be a part of cell component. AT5G51550, AT1G61640, AT4G31790, AT1G21840, AT3G55510, AT5G56580, AT3G14020, AT1G70530, AT2G31400 were found to be involved with biological process. Figure 6 shows a similar Venn diagram for *C* subunit. The diagram unveils that there are no genes present in all stress conditions in *C* subunit. Furthermore, there is no gene common in an intersection of any four, five, or six stresses. Unlike the previous two subunits, the majority of the genes are unique in their respective stresses. Not all intersections of any three stresses has common genes. For example, H $\cap$  O $\cap$ S, C $\cap$ D $\cap$ G, and H $\cap$ O $\cap$ D have 3 (AT5G11690, AT4G29910, AT5G05990), 11 (AT1G80690, AT4G32190, AT3G57330, AT1G28400, AT1G34340, AT1G51060, AT1G52310, AT3G25040, AT2G39700, AT2G19760, AT1G28110), and 1 (AT1G80720) common genes, respectively. But W $\cap$  O $\cap$  S, W $\cap$  O $\cap$  H, W $\cap$  O $\cap$  D and many such intersections do not have any common genes. AT5G11690, AT4G29910, AT5G05990 were found to be part cell component. Among the 11 genes, five genes: AT3G57330, AT1G34340, AT1G52310, AT3G25040, AT2G39700 were over-represented with the term ’membrane’. AT1G80720 is involved with mitochondrial matrix. Similarly, the count of all such 127 combinations of intersection can be identified from these 7-set Venn diagrams.

We performed GO analysis using the DAVID tool^32^ to interpret functional roles and the terms enriched with those in drought and salt conditions. Drought and salt genes in *A* group do not have any common genes while in *B* and *C* groups they have 38 genes and 22 genes in common respectively. GO analysis shows that 22 genes common to Drought and Salt are cellular components and enriched with the terms ‘small nucleolar ribonucleoprotein complex’ means they are involved with the spliceosome KEGG pathway, whilst the 38 genes are not enriched with any term. However, unique genes (288) in drought in subunit *A*, in salt are enriched with pentatricopeptide repeat (PPR), subunit *C* in drought genes (998) involved with ribonucleoprotein (translation). Genes unique to salt in *B* subunit (993) are involved with the ethylene signaling pathway. In drought, across the genes in *A*, *B*, *C* subunit genes (5855) most of the genes are involved with chloroplast functions while genes in salt across the *A*, *B*, *C* subunit genes (28) are involved with mitochondrial functions. Identification of common and unique genes followed by their GO term analysis on the genes has led to the pinpointing the function associated with the gene set. Most of the genes were involved with cellular components.

**Figure 7:**
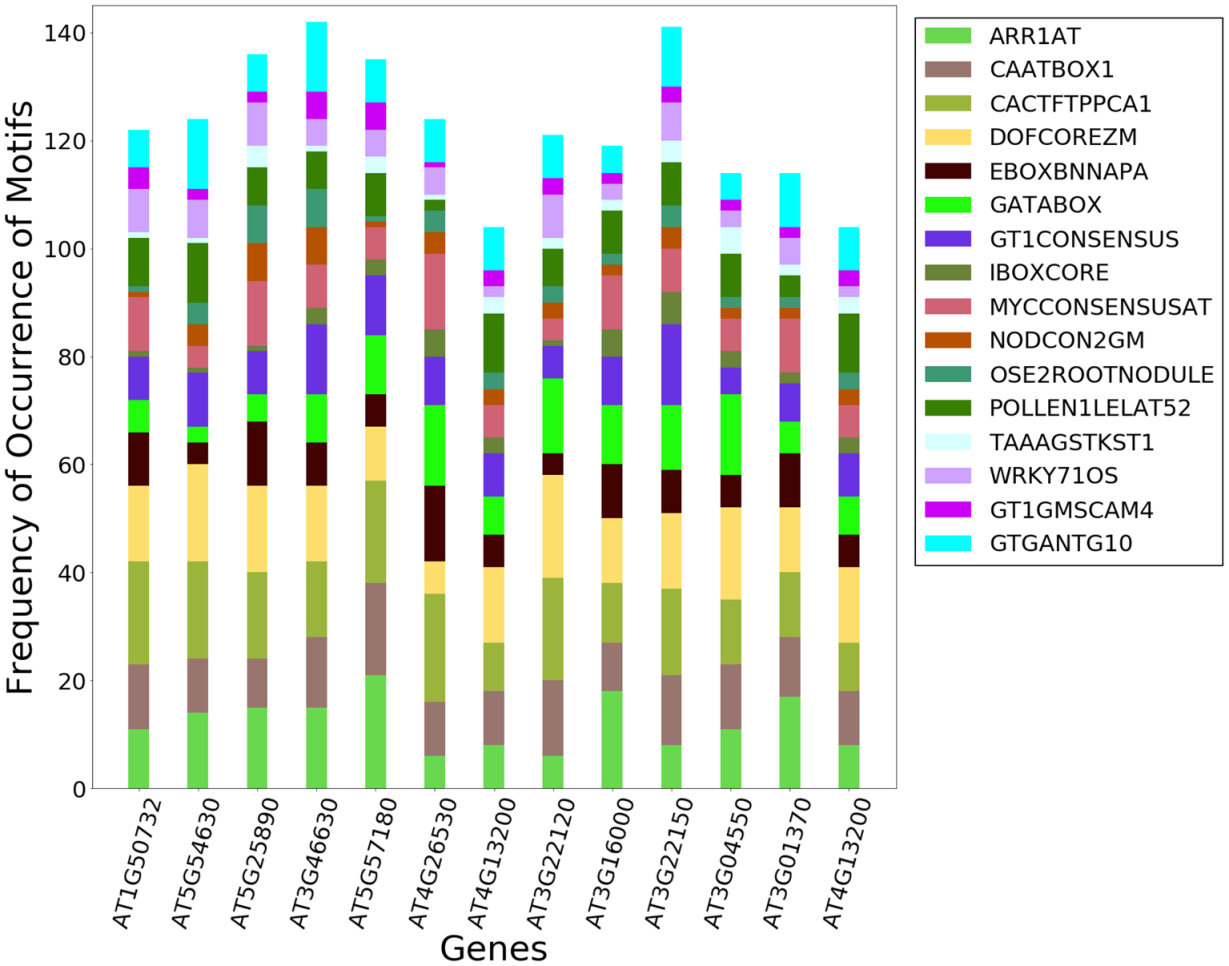
Motif analysis results conducted on 13 hub genes identified using graph analysis. Row-stacked barplot represents the absolute frequency of occurrence of motif in 13 genes. Motif analysis reveals the presence of 16 motifs in 13 genes.

### Motif analysis on hub genes reveals 16 common CREs

Generally, it is believed that genes co-expressing together are likely to contain a set of over-represented motifs within their promoters that lead to similar expression patterns. Numerous in-silico techniques have been developed to identify such motifs. Here we used the PLACE database to identify various CREs enriched within the promoter of those co-expressed genes. Motif analysis was done on the 13 genes having the high centrality scores in the network after web scraping data from Cress Express. Figure 7 shows the motif analysis conducted on 13 genes identified common across all subunits and having high CCS. Figure 7 illustrates the column stacked bar plot of the frequency of occurrence of motifs in 13 genes. The frequencies are shown in contrast to the 16 functions identified during motif analysis.

#### *CREs* and their role

Evaluation of 1-kb upstream promoter regions of 13 genes in which we found 16 motifs common across all the genes. Promoter sequences of genes exhibited the presence of several abiotic stress and biotic stress-responsive *cis*-elements. These motifs are: ARR1AT (5′-NGATT-3′), CAATBOX1 (5′-CAAT-3′), CACTFTPPCA1 (5′-CACGTG-3′), DOFCOREZM (5′-AAAG-3′), EBOXBNNAPA (5′-CANNTG-3′), GATABOX (5′-GATA-3′), GT1CONSENSUS (5′-GRWAAW-3′), GT1GMSCAM4 (5′-GAAAAA-3′), GTGANTG10 (5′- GTGA-3′), IBOXCORE (5′-GATAA-3′), MYCCONSENSUSAT, (5′-CANNTG-3′), NODCON2GM (5′-CTCTT-3′), OSE2ROOTNODULE (5′-CTCTT-3′), POLLEN1LELAT52 (AGAAA), TAAAGSTKST1 (5′-TAAAG-3′), WRKY71OS (5′-TGAC-3′). The frequency of occurrence of these CREs is depicted in Fig. 7 and their numbers are given in Supplementary Table S2. Among these 16 CREs, CACTFTPPCA1, is the most abundant of all CRE, having a total frequency of occurrence of 194 in the 1-kb upstream region of each gene followed by DOFCOREZM, ARR1AT, CAATBOX1 with 180, 158, and 150 respectively. CACTFTPPCA1, this tetranucleotide motif is known to regulate mesophyll-specific gene expression in plants^34^. We have found the highest occurrence of this motif in genes under study which reveals the importance of this motif in the regulation of stress responses in PP2A genes with the stress-responsive genes. DOFCOREZM is a known binding site of Dof (DNA binding with one Zn finger) family of TFs, which are specific DNA-binding proteins associated with the expression of multiple genes in plants^35^. Dof TFs are involved in differential regulation of a variety of promoters in various plant processes such as development, seed-specific expression and abiotic and biotic stress responses. This motif is the second most abundant motif across the genes. ARR1AT (ARR1-binding element) in *Arabidopsis* is a cytokinin response regulator which acts as transcriptional activators^36^. CAATBOX1, mediates the tissue-specific promoter activity of the pea legumin gene LegA in tobacco^37^. GATABOX, is an important motif in light regulation and tissue-specific expressions^38^. GT1CONSENSUS consensus sequence is a binding site for GT-1 in many light-regulated genes^39^. GTGANTG10, is a GTGA motif found in the promoter of tobacco late pollen genes^40^. MYCCONSENSUSAT recognition site is found in the promoters of the dehydration-responsive gene rd22 and several other genes in Arabidopsis. It is also known as cold responsive element^41^. POLLEN1LELAT52 responsible for pollen-specific activation in plants such as tomato^42^. IBOXCORE is a conserved sequence found upstream of light-regulated genes of both monocots and dicots^43^. WRKY71OS is a binding site of rice WRKY71, a transcriptional repressor of the gibberellin signaling pathway; WRKY proteins bind specifically to TGAC-containing W box elements within the Pathogenesis-Related 10 (PR-10) genes^44^. Family members of WRKY transcription factors are known to be involved in the regulation of pathogen defence responses, abiotic stress responses such as heat and salinity^45^, and plant growth and development as well. NODCON2GM One of two putative nodulin consensus sequences^46^. OSE2ROOTNODULE One of the consensus sequence motifs of organ-specific elements (OSE) characteristic of the promoters activated in infected cells of root nodules^47^. TAAAGSTKST1 is known for guard cell-specific gene expression^48^. EBOXBNNAPA is an ABA response element^49^.

**Figure 8:**
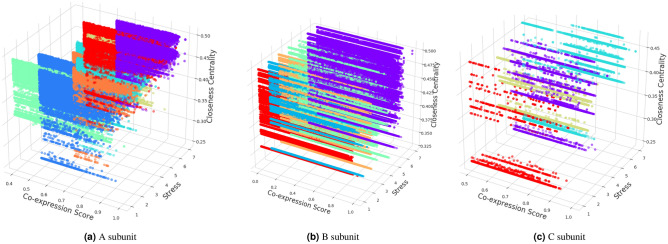
3D Scatter plot illustrating the clustering results. Three dimensions are co-expression value, stress condition, and closeness centrality. Colours represent different clusters. Genes in A, B, and C subunits are grouped into 7, 5, and 4 clusters, respectively.

### Clustering results and module identification

Figure 8a–c illustrate the results of *K*-means clustering on *A*, *B*, and *C* subunits, respectively, conducted in Section Clustering. As shown in the Fig. 8, the clustering is performed on three dimensions; stress condition, co-expression value, and closeness centrality of genes from graph analysis. Each gene id is represented using circles and clusters are distinguished with different colours. Stresses are represented in numbers from 1 to 7 in the following order: Cold, Heat, Genotoxic, Drought, Salt, Osmotic, and Wounding. Figure 8 shows that due to the large number of genes in each subunit, the visual interpretation of clusters is challenging; therefore, we refer to the cluster results used for the visualisation. As discussed in Section Clustering, based on the silhouette scores, the genes in *A*, *B*, and *C* subunits are grouped into 7, 5, and 4 clusters, respectively. Based on the unique occurrences (different combinations of three dimensions), one gene may occur more than once. Therefore, the total number of data points in *A*, *B*, and *C* subunits are 285,933, 625,511, and 40,847, respectively. *A* subunit has total five genes forming the subunit further combined with seven stress conditions; thus a gene can appear maximum 35 times in *A* subunit and similarly, 70 and 14 times in *B* and *C* subunit; increasing the total number of gene points in high-dimensional space. Based on our cluster results, we find that the gene id AT3G46690 is present in maximum combinations in *A* subunit i.e. 27 times followed by 53 genes with an occurrence of 26 times. Interestingly, we also find 327 such genes which are present exactly once in *A* subunit (only one combination out of 35). Out of these 327 genes, only five (AT5G59870, AT3G57220, AT1G28510, AT5G43640, AT5G50830) are present in cluster 2. Whereas 183 and 139 genes are present in clusters 4 and 5. We look at the properties of these genes and find that five genes identified in cluster 2 are present (1) only once in the dataset, (2) present in drought stress condition, (3) have same CCS of 0.331508, and (4) have a negligible difference in co-expression values (maximum 0.1). Similarly, the genes identified in clusters 4 and 5 are present in genotoxic and wounding stresses with a significant variation in their co-expression values and CCS. On manual inspection in *A* subunit results, we observe that due to the large input size and multiple occurrences of same probe id in same stress but different genes ($A_1$ to $A_5$), the targeted stress is the most influential feature for clustering; further indicating the number of optimal clusters in *A* subunit. In *B* subunit, i.e. Fig. 8b, the maximum number of genes in a cluster are 170,930 with their multiple occurrences while unique genes are 11,959. Based on our manual inspection, we find that the majority of these 11,959 unique genes are present in drought and salt stresses. This insight reveals that there are several genes in salt and drought stress conditions that have similar behaviour in-terms of CCS and slight variations in their co-expression values. Some of these genes are AT3G03170, AT1G60800, AT2G30520, AT5G32450, AT4G37080, AT5G33280, AT5G37680, AT5G17760, AT4G37300, AT4G34230. In *C* subunit depicted in Fig. 8c, the minimum number of genes (with multiple occurrences) grouped in a cluster are 1524. Out of 10,276 genes, 155 are present only once and interestingly, they all are present in heat stress condition with the same CCS of 0.252578. Some of these genes are AT2G28370, AT2G03890, AT5G01460, AT3G01390, AT1G66750, AT2G20190, AT5G24890, AT3G60080, AT5G09920, AT1G04010, AT5G13760, AT1G29060, AT3G58760, AT3G56850, AT3G09980. Unlike *A* subunit, in *C* subunit, stress is not the primary factor for clustering since we also find examples of gene ids which appear on different stresses but end up in the same cluster. For example, AT4G13950 appears on both cold and heat stresses with the same CCS but different Co-expression values. Still, based on the similarity in other two dimensions than stress condition, both occurrences of these genes are clustered in one group. Our clustering results reveal that the largest cluster is formed by the genes which appear (one or multiple times) in osmotic and wounding conditions. We treat these clusters as modules for identifying the genes appearing together the most. During clustering analysis, we observe that since the gene ids selected for graph analysis had co-expression value above mean, hence default on the higher side; relatively higher CCS in comparison to many other genes. Therefore, the clustering is more inclined towards the stress conditions. Whenever data points (genes with multiple occurrences) are not dense (unlike subunit *A*), genes appearing in different stress conditions are also grouped in the same cluster.

### Unique CREs

Besides the most abundant CREs across the genes, we also found unique CREs constrained to a particular gene only. We found 28 CREs occurring only once in nine genes. These CREs are presented in Supplementary Table S3.

## Materials and methods

### Experimental datasets

Publicly available co-expression datasets deposited in the CressExpress^50^ for *Arabidopsis thaliana* were downloaded and processed (Supplementary Tables S1A, S1B, and S1C). The data included seven environmental conditions cold (GSE5621), Osmotic (GSE5622), Salt (GSE5623), Drought (GSE624), Genotoxic (GSE625), Wounding (GSE627), Heat (GSE628) which was generated using an Affymetrix platforms. The number of experiments given for stress conditions: 24 for cold, 24 for genotoxic, 28 for wounding, 24 for osmotic, 24 for salt, 32 for heat, 28 for drought. The total number of collected genes across all experiments for subunit *A* was 1,01,315; 39,280 for subunit *B*; 1,23,025 for subunit *C* (Table 2). When the similarity scores between all gene pairs have been determined, a cutoff is applied to select the gene pairs that should be connected in the network.

**Table 2:** The total number of collected genes across the experiments in stress conditions for various subunits of PP2A. Columns represents the count of unique and common genes across A, B, and C subunits. A $^{\prime }$, B $^{\prime }$, and C $^{\prime }$ represent the numbers of genes which are unique in these subunits but not present in other respective subunits. Intersection ($\cap$) and Union ($\cup$) are shown for overlapping genes.

		Stress conditions						
		Cold	Drought	Genotoxic	Heat	Osmotic	Salt	Wounding
Total	A	14,700	9114	16,219	9114	9114	13,326	16,219
	B	18,606	17,751	17,872	18,247	16,774	16,441	17,355
	C	1324	10,768	11,414	403	1270	504	13,604
Set-wise Distribution	A $^{\prime }$	358	11	197	752	326	434	1913
	B $^{\prime }$	4201	4830	1774	9571	7558	3514	1733
	C $^{\prime }$	10	7	14	5	201	23	52
	A $\cap$ B	13,129	2161	4710	8280	8152	12,458	2120
	A $\cap$ C	38	1	12	2	5	12	50
	B $\cap$ C	101	3819	88	316	433	47	1366
	A $\cap$ B $\cap$ C	1175	6941	11,300	80	631	422	12,136
	A $\cup$ B $\cup$ C union	**19,012**	**17,770**	**18,095**	**19,006**	**17,306**	**16,910**	**19,370**

**Figure 9:**
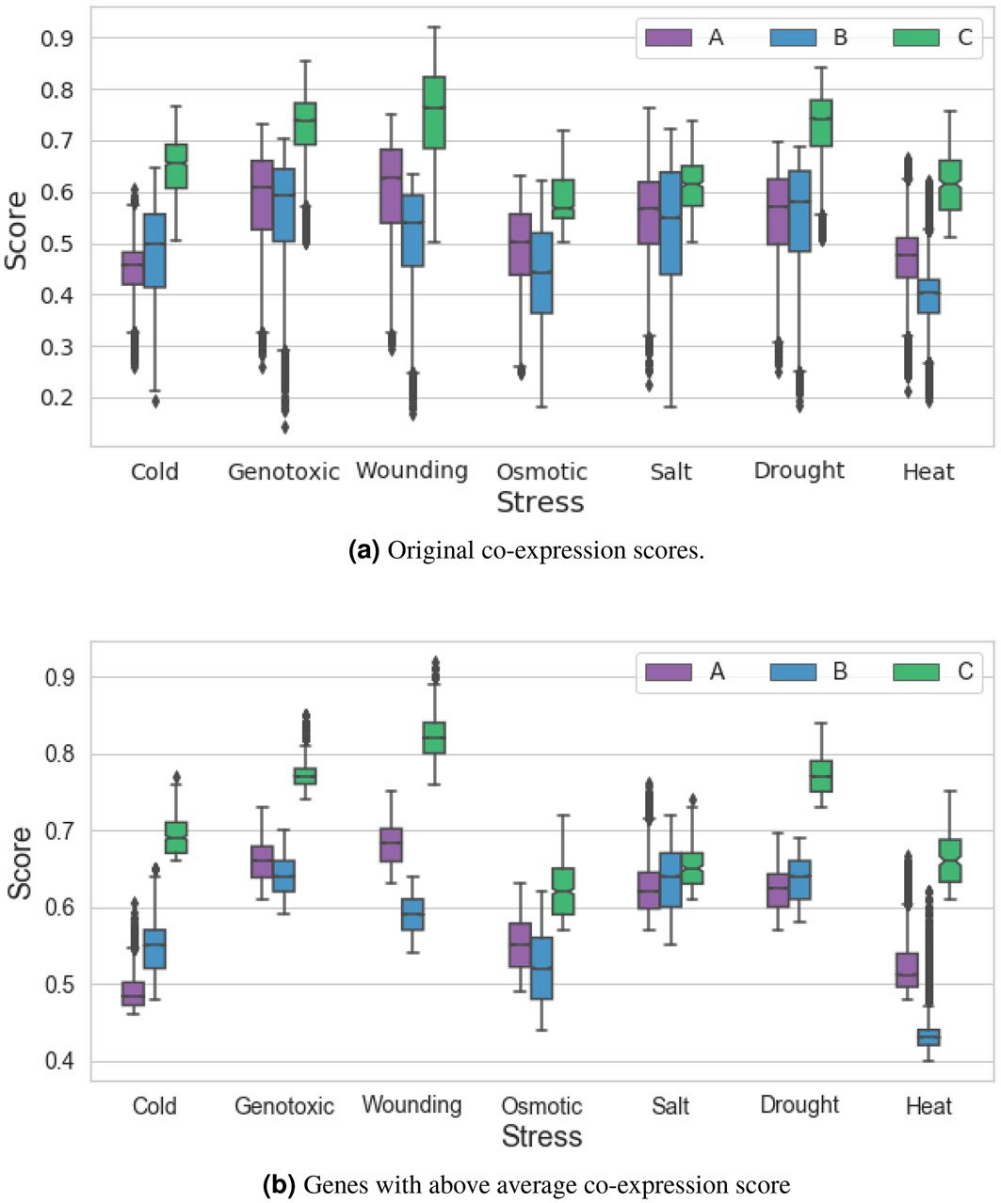
Co-expression correlation distribution of genes in A, B, and C subunits across all stresses.

### Co-expression network construction and parameters

Numerous methods have been developed until now for constructing gene co-expression networks in plants. To construct gene co-expression networks, two approaches need to be considered: (1) calculating co-expression measure, where any one of the common measures such as Pearson’s correlation coefficient, Mutual Information, Spearman’s rank correlation coefficient, and Euclidean distance is selected, and a similarity score can be calculated for each pair of genes using that measure; (2) selecting a significant threshold, after selecting an appropriate co-expression measure, a similarity score is calculated for each pair of genes using that measure. Then, a threshold is determined in order to select gene pairs showing a similarity score higher than the threshold are considered to have a significant co-expression relationship, and those genes in the form of nodes (vertex) are connected by an edge in the co-expression network.

In the Cressexpress database, Pearson’s correlation coefficients (PCCs) of gene expression patterns are used as a measure of gene co-expression. PCC is the most popular co-expression measure used in constructing gene co-expression networks usually measures correlation (linear dependence) between two variables^51^. Pearson’s correlation coefficient takes a value between − 1 and 1 where absolute values close to 1 shows a strong correlation. The positive values correspond to an activation mechanism where the expression of one gene increases with the increase in the expression of its co-expressed gene and vice versa. When the expression value of one gene decreases with the increase in the expression of its co-expressed gene, it corresponds to an underlying suppression mechanism and would have a negative correlation. There are two disadvantages for Pearson correlation measure: it can only detect linear relationships and it is sensitive to outliers. Moreover, the Pearson correlation assumes that the gene expression data follow a normal distribution.

Figure 9(A) shows the distribution of co-expression average correlation score of all genes present in each subunit across all seven stresses. We use standard box plot representation to show the distribution where X-axis represents the stresses, y-axis represents the average score, and legends represent the subunits (annotated with different colours). Figure 9(A) reveals that *C* subunit has a higher average co-expression score in all stresses followed by *B* and then *A* subunit genes. The box plots also reveal the usual behaviour pattern in similar genes; e.g. salt and drought. Based on existing literature, we also plot the distribution graph for the top 50 genes with the highest co-expression score. In previous studies, mostly co-expression data is measured by either correlation coefficients or mutual information measures^52^. However, Supplementary Fig. S1 reveals that the selection of the top 50 genes brings bias in the gene analysis since the co-expression is mapped in a very short span of scores. Furthermore, the analysis of only the top 50 genes will not provide any actionable insights since they have similar scores and may not be present in other stresses or subunits. Selection of top 50 genes also loses the essence of usual behaviour pattern. Furthermore, the top 50 genes present in salt and drought do not have similar distribution unlike Fig. 9(A). To address the challenge of existing literature, we proposed to analyse all those genes whose average co-expression value is greater or equal to the mean of overall distribution. Figure 9(B) shows the distribution (box plots) of genes with a co-expression value above average. Figure 9(B) illustrates this distribution across all stresses and sub-units. The proposed hypothesis addresses two major gaps of existing literature: (1) The genes identified for further analysis do not have scores within a short span and hence shows significant cohesion and distortion in genes behaviour; and (2) The co-expression value distribution has the same behaviour as original genes yet reducing the background noise in the analysis.

We use these genes for generating the gene network to conduct graph analysis and identify influential genes. Without imposing a selection criterion, every gene would be connected to every other with a weighted edge (− 1.0 $\le$ p $\le$ 1.0). The resulting graphs would be overly large and dominated by uninformative edges. Hence, this initial threshold (co-expression value above mean score) is used to define a starting point for subsequent analysis, and nodes with no connections above the selected threshold are removed from the graph. The size of the graph produced is, therefore, dependent on the threshold level selected. PCC is the most common and reliable measure of co-expression than any other measure to reflect the linear correlation between any two genes. We selected the Cressexpress tool for this reason for our analysis. The higher PCC scores show the closer relationships between the genes. For data analysis, firstly using pandas and excel reader the selected genes were imported in a python list and transformed into a triplet of <source, target, weight>. The *Source* represents the gene id, *Target* represents the unique subunit ($A_1$ to $A_5$, $B_1$ to $B_{10}$ and $C_1$, $C_2$), and *Weight* represents the individual co-expression value for each part of the subunits. From the above matrix, the co-expression network was formed using Gephi (open-source network analysis and visualisation software package) for every stress condition and all the subunits of PP2A. Initially, for the analysis stress condition data on different subunits were considered separately but were then merged afterwards to get a holistic view.

### Network measures and gene association

To identify the relationship within genes, stresses and subunits, we create a network of gene ids and associated entities. The network consists of two sets gene IDs $\{G_{k} \mid 1\le k\le n; \text { n is the number of genes present in a subunit}\}$ and Stress_Unit $\{SU_{i,j} \mid 1\le i\le 7 \text { and j is the number of subunits}\}$. These sets form a bipartite graph since no genes are connected with each other but connected with a set of regulatory subunits within stress. Since the aim is to capture the most influential and hub genes, we use the closeness centrality metric to represent the network in each subunit setting. In a graph *G*(*V*, *E*), the closeness centrality of a node $x \in V$ tells the mean distance from *x* to every other node $y \in V$ where $x \ne y$ and can be computed as

1 $$\begin{aligned} C_x = \frac{n}{\sum _y d_{x,y}}\end{aligned}$$

In other words, it reveals the connectivity and reachability of a node. A node with a high closeness centrality has the shortest distance from other nodes. In the context of a gene network, the closeness centrality is referred to as the occurrence of a gene in different stresses and in different subunit genes. We create such network for each of *A*, *B*, and *C* subunits and later identify the influential genes across all subunits by taking an intersection of individual subunits. We perform motif analysis on the genes that are common across all subunits irrespective of the stress.

### Clustering

After performing network analysis on gene data, each gene id is represented as a triplet of < stress, closeness centrality, co-expression score >. We represent each gene as a data point in these three dimensions for clustering. Clustering is an unsupervised learning mechanism that divides the input data points into a number of groups called clusters and investigates the similarity patterns among them. It aims to identify the group of data points that are highly similar to each other and very dissimilar to the instances present in other groups. Thus, clustering also helps in identifying the modules. We apply clustering on the genes acquired from graph analysis and validate them against their natural characteristics. We used the *K*-means clustering algorithm to fit our unsupervised model. Since the number of *K* clusters in *K*-means is user-generated (i.e. pre-determined) and sometimes can lead to poor clustering if not selected carefully, we use Silhouette Score^53^ to identify the number of optimal clusters. The silhouette score is a well-known and commonly practised metric to identify the value of *K*. It uses the concept of mean inter-cluster similarity and mean intra-cluster similarity and ranges from − 1 to 1. It iteratively computes these distances/similarities for different values of *K*. We set the range of finding optimal clusters between 2 and 9 since two clusters are minimum requirement for the proposed analysis. The upper range is selected slightly above the number of stress conditions in our dataset. For each value of *K*, the model runs 10 times and selects *K* random data points as initial centroids. We use *K*-means++ as the method of initialisation which selects initial centroids uniformly at random to increase the convergence rate ?. Further, for every value *K*, the model runs for maximum 300 iterations. To declare the convergence, we set relative tolerance as $1\mathrm {e}{-4}$ (0.0001) which defines the difference between cluster centroids for two consecutive iterations. The aim is to increase the intra-cluster similarity (cohesion) and decrease the inter-cluster similarity (distortion- representing the separation from genes in other clusters). A larger Silhouette value indicates that clusters are significant.

**Figure 10:**
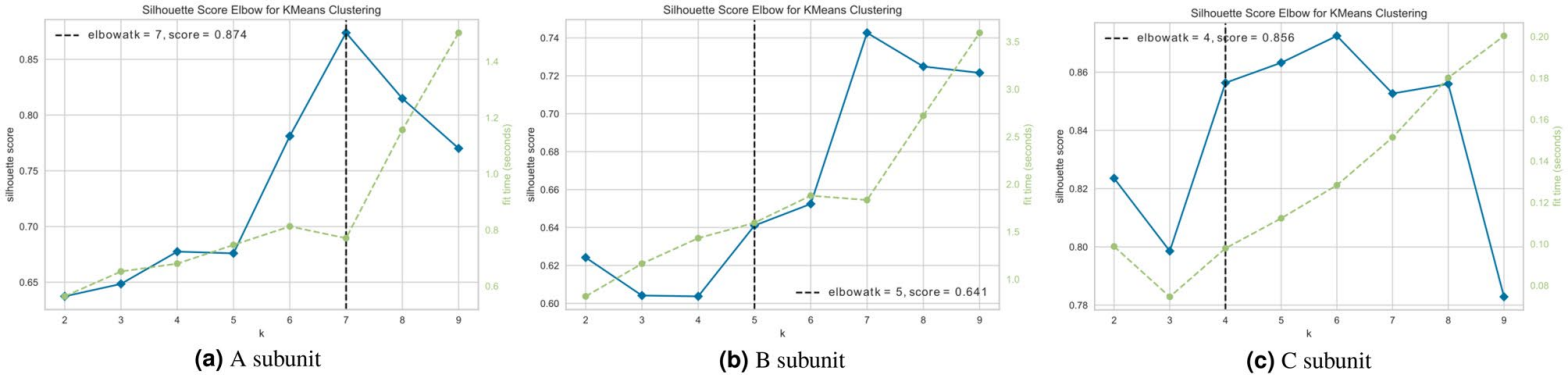
Silhouette score elbow curve showing optimal K for clustering. The silhouette score identifies the optimal value of clusters in K-means clustering. It ranges from − 1 to 1 with an aim to increase the intra-cluster similarity and decrease the inter-cluster similarity. A larger Silhouette value indicates that clusters are significant.

Figure 10a–c illustrate the Silhouette score elbow curve for genes present in *A*, *B*, and *C* subunits, respectively. Figure 10a shows that the optimal value of *K* for genes in *A* subunit is 7. Similarly, Fig. 10b shows that the optimal number of clusters in *B* subunit is 5. Even though *K* = 6, 7, and 8 have higher Silhouette scores than *K* = 5, those are bad picks for the optimal value of *K* due to wide fluctuation in the score. The fit time represents the time taken (in seconds) to fit the *K*-means model on the given data. Similar to Fig. 10b,c shows that the optimal number of clusters in *C* subunit is 4. The time graph shows that the algorithm took minimum time to fit the model for *K* = 4. While *K* = 5, 6, 7 have a higher scores than *K* = 4, there is a lot of fluctuation in score and furthermore there is an instant decline in score after increasing the value of *K* slightly. *K*-means clustering generates well-shaped hard clusters; one instance belongs to only one cluster and no two clusters have overlapping instances. However, in the context of gene clustering, one gene has multiple occurrences in the input dataset and hence can be present in multiple clusters. For example, if a gene id is present in multiple stresses in the same subunit then it will have the same centrality score but two different stress target and co-expression values. Therefore, based on the difference between these two dimensions, the same gene might appear in different clusters.

### Analysis of *cis*-regulatory elements (CREs) on hub genes

We performed *cis*-element analysis following co-expression network analysis^21^. Further, genes used to identify conserved and unique motifs. We aimed to take *in-silico* methods to investigate candidate *cis*-regulatory elements (CREs) associated with PP2A and stress-regulated gene expression. For this, the promoter sequences of 1 kbp upstream from the transcription start site for each gene from under consideration were retrieved from TAIR^54^ database. PLACE^55^ was used for scanning and identification of motifs in promoter regions of hub genes. PLACE database gives information on motifs already reported from previously published literature.

Motifs present in the genes were extracted by ‘Web Scraping’ the PLACE Database. Web Scraping is a technique employed to extract large amounts of data from websites whereby the data is extracted and saved to a local file in the computer or to a database in table (spreadsheet) format. The 1000 bp upstream sequence of all the common genes was downloaded from the Cress Express website which acted as the input for the scraping code. The output of each gene was a table with the motif name, sequence, its position in the promoter region, and the site number.

This was given as the input in the ‘Binary Search’ with each row of the table corresponding to a list and the inputs with the same motif name, sequence, and site number but different locations in the upstream sequence were clubbed together and the corresponding frequency was calculated.

## Discussion

Due to climate abnormalities, crops encounter multiple abiotic and biotic stresses throughout their developmental spectrum, which severely affect their growth, productivity, quality and yield in the field conditions. Since continuous exposure to these environmental insults is destructive to crop production, the identification of potential target genes imparting tolerance to a variety of stresses is highly expedient. Simultaneous occurrences of drought and heat are well known to cause harmful effects to crop production as compared to other abiotic stresses occurring individually at different growth phases. Abiotic stress conditions are also directly linked with the biotic stresses. Drought, heat, and salt have a negative influence on the occurrence and spread of insects, pathogens and weeds^56^. These stresses are also known to alter plant physiology and defence responses, thereby affecting plant-pest interactions^57^. In this study, we employed various abiotic stresses microarray datasets (cold, drought, heat, osmotic, genotoxic) and biotic stress (wounding) from *Arabidopsis thaliana* available on databases for co-expression analysis. Our analysis unveiled 13 genes as the core stress-responsive genes modulating *Arabidopsis* responses to multiple abiotic or biotic stresses. These genes are candidate genes for improving *Arabidopsis* environmental stress tolerance, which has not been investigated in studies aimed at a single stress.

Gene co-expression networks have been known to understand biological problems in terms of gene networks. In this study, we constructed a co-expression network of PP2A with genes involved in various stress conditions for the identification of gene co-expression modules to reveal the relationships between gene expression and stress regulation. Gene co-expression analysis of PP2A genes with stress-responsive genes provides insights into the role of PP2A in both abiotic and biotic stress responses as the major CREs involved in stress responses are enriched within the promoters of these genes. Our data shows the occurrence of conserved motifs, which are putative CREs and are likely targets of transcriptional factors regulating PP2A gene expression during abiotic and biotic stress conditions. These motifs can be used to design the experimental verification of regulatory elements and the identification of corresponding TFs regulating PP2A gene expression in plants. By analysing co-expression networks constructed from gene expression data, we can identify a large number of unknown genes with a regulatory role in abiotic and biotic stresses. Till date, it has found great utility in improving our understanding of pathways that are regulated at the transcriptional level, such as abiotic and biotic stress responses, various cellular and developmental processes.

In summary, through this analysis, we identified 16 CREs/motifs commonly occurring in all the 13 genes and 28 unique motifs in them. The high abundance of these 16 motifs across all the 13 genes shows their role in regulating the expression of the PP2A genes containing them and responsible for cross-talk between the various stress conditions. These genes are chloroplast related genes. Studies also reveal that chloroplast retrogrades regulation of heat stress responses in plants^58,59^. The unique motifs present within the promoters of the genes might be responsible for driving high expression of these genes during simultaneous exposure to various abiotic and biotic stress under study. Further, laboratory research must be carried out to verify these results. Designing and development of inducible promoters responsive to stresses necessitates sequence identification and functional characterisation of various *cis*-acting regulatory elements/motifs during diverse stress conditions in plants^60,61^. We employed clustering analysis to break down the genes involved in stresses into different functional modules or clusters. Our clustering data reveals that stresses like salt and drought stresses have many genes in common according to the co-expression values. This is because plants’ response to salinity and drought are often similar. However, genes in Drought and salt have some genes common which are involved with the spliceosome meaning they take part in RNA splicing. Venn diagram shows which are specific to the stresses and genes which are overlapping across the multiple stresses. Through the Venn diagram, we got to know that in drought and wounding stress, *A* subunit has unique genes. Drought and salt in *A* subunit do not have any common genes while in *B* and *C* subunits they have 38 genes and 22 genes common. GO analysis on them shows that 22 genes common to Drought and Salt are cellular components of the cell and enriched with the terms ‘small nucleolar ribonucleoprotein complex’ means they are involved with the spliceosome KEGG pathway, whilst the 38 genes are not enriched with any term. However, unique genes (288) in drought in subunit *A*, salt are enriched with pentatricopeptide repeat (PPR), subunit *C* in drought genes (998) involved with ribonucleoprotein (translation). Genes unique to salt in *B* subunit (993) are involved with the ethylene signaling pathway. In drought, across the genes in *A*, *B*, *C* subunits (5855) most of the genes are involved with chloroplast functions while genes in salt across the *A*, *B*, *C* subunit genes (28) are involved with mitochondria.

We identified 13 genes as the most influential genes regarded as hub genes in the network which are the most connected genes common across all the subunits with the highest closeness centrality. CRE analysis on these genes had led us to the identification of some common and unique motifs. These highly associated common genes within the network of stress associated genes coexpressing with the *A*, *B*, *C* subunits of PP2A could be used for genetic improvement and to study molecular regulatory mechanisms of plants and their orthologues can be used for genetic improvement in other crops. These candidate genes can be used as a major route to develop crops tolerant to abiotic stress, including heat, salt, osmotic, drought, cold, genotoxic stress and biotic stress such as wounding.

## Conclusion

PP2A is known for its crucial role in a diverse range of processes in the cell, including abiotic and biotic stress responses. Here, we performed a co-expression driven motif analysis on various genes involved in different stresses with PP2A genes from *Arabidopsis thaliana*. The analysis reveals 16 motifs commonly occurring in all the 13 hub genes identified and some unique motifs. The analysis showed the importance of these motifs in the regulation of these stresses in *A. thaliana*.

## Supplementary information

Supplementary Information 1.

Supplementary Information 2.

Supplementary Information 3.

Supplementary Information 4.

Supplementary Information 5.

Supplementary Information 6.

## Data Availability

The data generated/analysed during the study are available in the supplementary files.. The datasets collected and analysed for this study can be found in the Google Drive Repository. Link to the dataset: https://drive.google.com/drive/folders/1FPzD2_Xk-k0Hzjzem67xpnQujPuycxuj?usp=sharing
