# Supplementary material for: Co-expression network analysis of protein phosphatase 2A (PP2A) genes with stress-responsive genes in Arabidopsis thaliana reveals 13 key regulators
Source: Sci Rep. 2020 Dec 8;10:21480. doi: 10.1038/s41598-020-77746-z (PMC7722862; doi:10.1038/s41598-020-77746-z)
Supplement: Supplementary file 1 — Supplementary Information 1. [file 41598_2020_77746_MOESM1_ESM.pdf]

Co-expression Network Analysis of Protein Phosphatase 2A (PP2A) Genes with Stress-Responsive Genes in Arabidopsis thaliana Reveals 13 Key Regulators

Zaiba Hasan Khan, Swati Agarwal, Atul Rai, Mounil Binal Memaya, Sandhya Mehrotra, and Rajesh Mehrotra

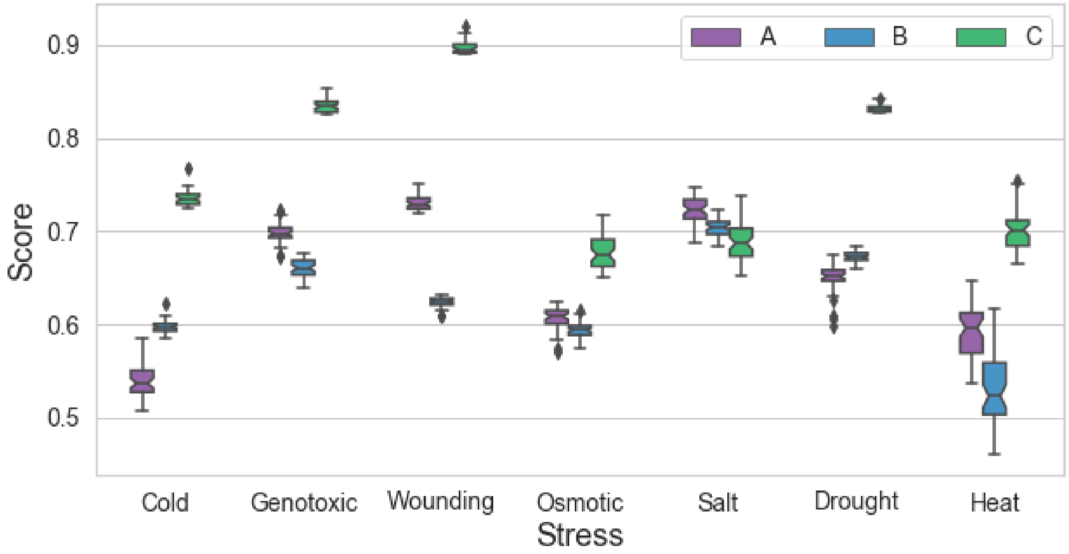

Supplementary Figure S1: Box-plot representation of co-expression value distribution among top 50 Genes present in A, B, and C subunits.

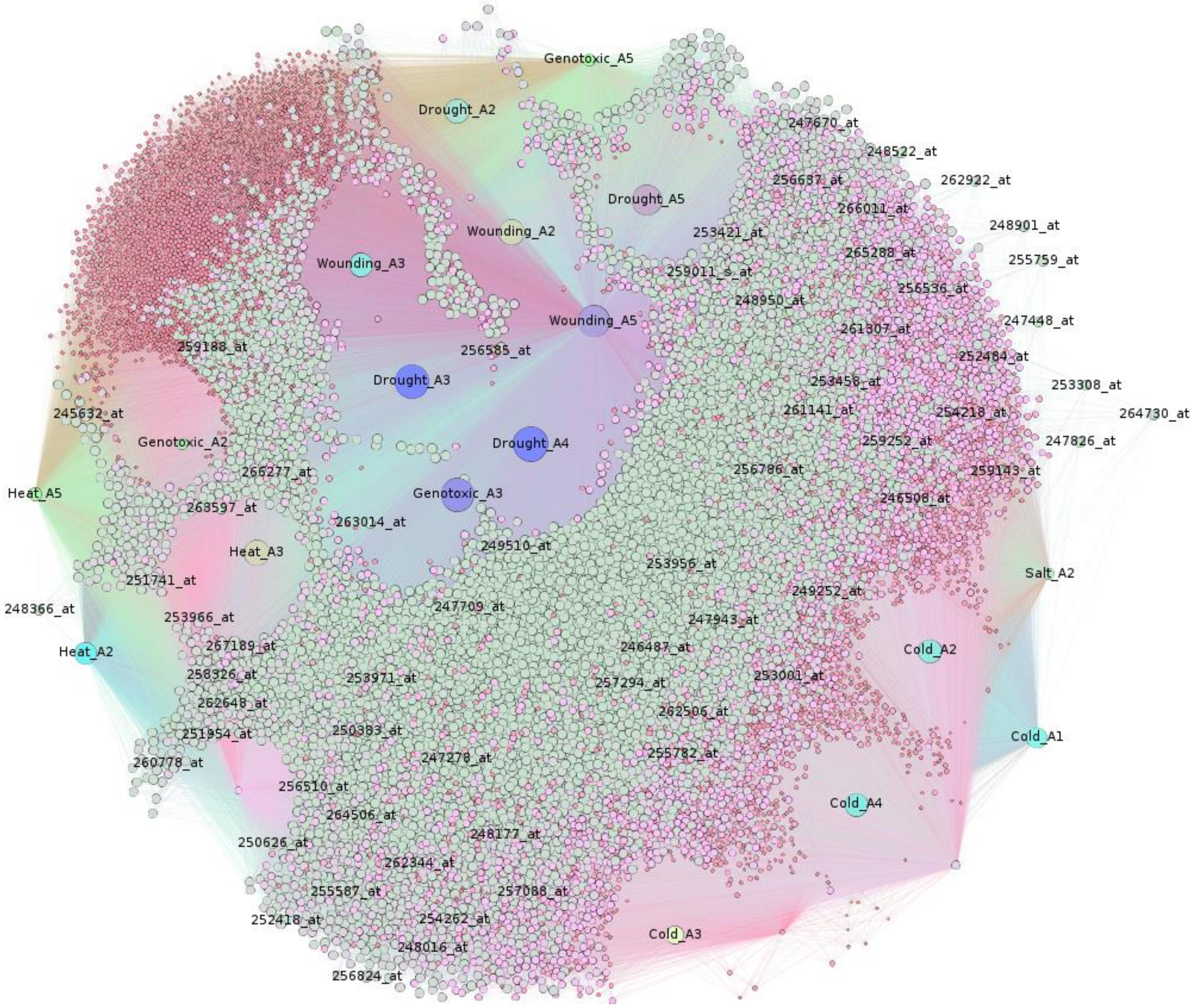

Supplementary Figure S2: Graph analysis of genes present in A subunit across all stresses. Size of the node represents the relative closeness centrality score. Colour demonstrates the similar nodes.

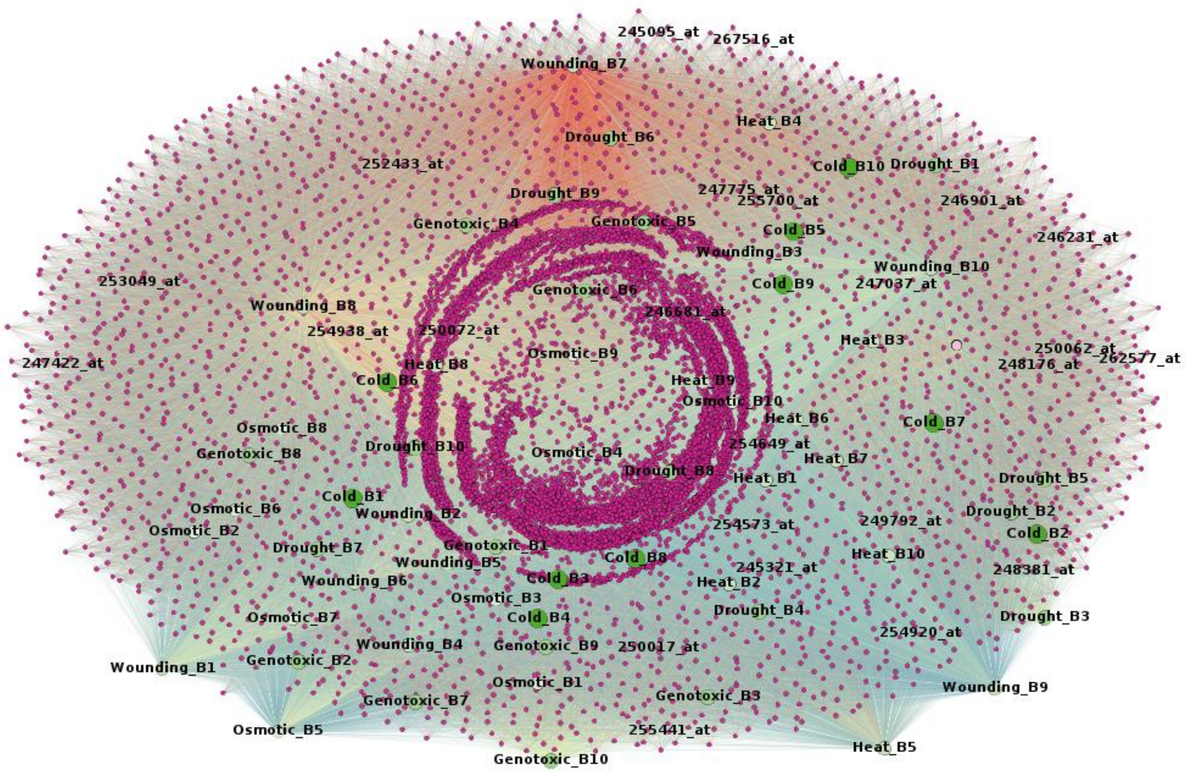

Supplementary Figure S3: Graph analysis of genes present in B subunit across all stresses. Size of the node represents the relative closeness centrality score. Colour demonstrates the similar nodes.
